# CT-Negative Fish Bone Impaction in the Pyriform Sinus Requiring Operative Removal: A Case Report

**DOI:** 10.7759/cureus.107701

**Published:** 2026-04-25

**Authors:** Madison Lather, Bianca McAravey, Austin D Layton, Lily House, Michael Burchett

**Affiliations:** 1 Department of Surgery, Rocky Vista University College of Osteopathic Medicine, Billings, USA; 2 Department of Surgery, Marian University College of Osteopathic Medicine, Indianapolis, USA; 3 Department of General Surgery, Mercyone Genesis Health System, Davenport, USA

**Keywords:** diagnostic imaging, fish bone, foreign body, globus, odynophagia, otolaryngology

## Abstract

Fish bone impaction in the oropharynx is a common cause of foreign body sensation and odynophagia. While computed tomography (CT) has the highest sensitivity and specificity among radiologic imaging modalities in the detection of fish bone foreign bodies, false negatives still occur. This case demonstrates the critical importance of clinical judgement and further evaluation when foreign body symptoms persist despite negative imaging findings.

A 67-year-old woman presented to the emergency department (ED) twice within 24 hours with persistent odynophagia and foreign body sensation after eating catfish. CT imaging demonstrated no foreign body on both occasions. Oral examination and flexible endoscopy under anesthesia were subsequently performed due to persistent symptoms. Inspection of the oral cavity revealed a 4-cm fish bone embedded in the left pyriform sinus. The bone was extracted without difficulty. The patient recovered without complication and had immediate resolution of her symptoms.

This case highlights that fish bones can be radiologically occult and that persistent symptoms of odynophagia and foreign body sensation following fish ingestion warrant further evaluation regardless of negative imaging findings. Clinical judgement should supersede imaging results in suspected foreign body cases, as delayed removal increases complication risk (esophageal laceration and perforation, ulceration, mediastinitis, as well as abscess formation). Emergency physicians and otolaryngologists should maintain a high index of suspicion when clinical presentation strongly suggests fish bone foreign body impaction despite negative imaging findings.

## Introduction

Foreign body ingestion is a common emergency department (ED) presentation [1-5]. Among ingested foreign bodies, fish bones are the most frequently encountered in adults [2] and account for over 84% of accidental ingestions [6]. Most fish bones are lodged in the oral cavity, or oropharynx [2-4], and common sites of impaction include the base of the tongue, palatine tonsils, vallecula, and pyriform sinus [4,6,7]. Fish bone impaction can cause symptoms, such as foreign body sensation, odynophagia, and dysphagia, and may require otolaryngologic intervention due to the risk of migration and surrounding tissue injury. If not promptly identified and removed, retained fish bones may lead to complications, including infection, abscess formation, or penetration into adjacent structures [8-10].

Computed tomography (CT) is a commonly used imaging modality for foreign body identification and has high sensitivity and specificity for fish bone foreign body detection [2,11-13]. However, radiologic evaluation alone in fish bone foreign body diagnosis can produce false negative results [2].

We present the case of a symptomatic patient with a retained fish bone foreign body who was discharged from the ED on multiple occasions due to negative CT findings. This case highlights the potential for retained foreign bodies to evade initial imaging and emphasizes the importance of a thorough clinical evaluation and maintaining a high degree of clinical suspicion for foreign body impaction in patients with persistent symptoms.

## Case presentation

**Figure 1:**
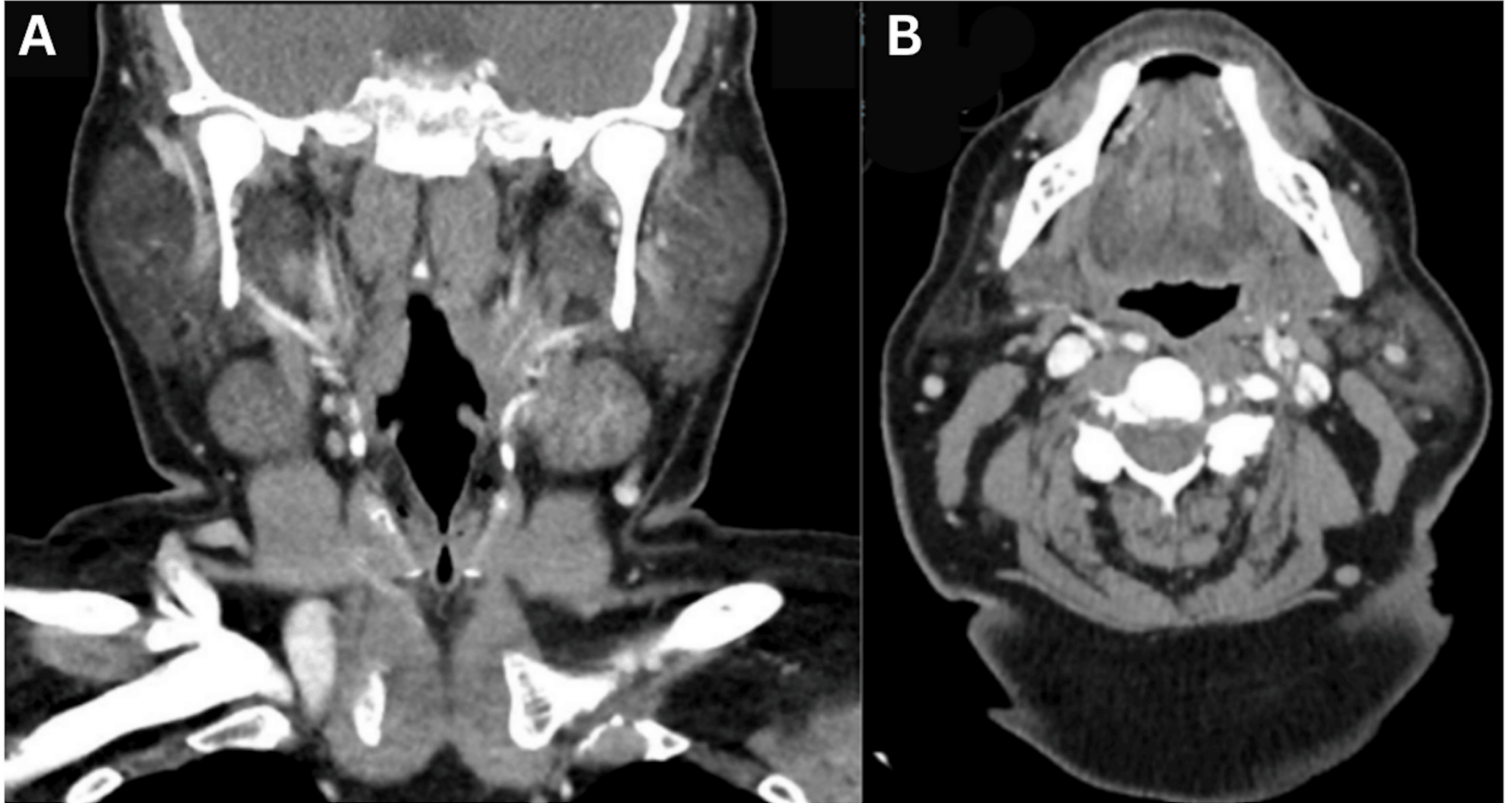
Neck CT with contrast in a patient with a suspected foreign body after consumption of catfish. No foreign body was identified on imaging as demonstrated above. A) Coronal view neck CT with contrast. B) Transverse view neck CT with contrast.

**Figure 2:**
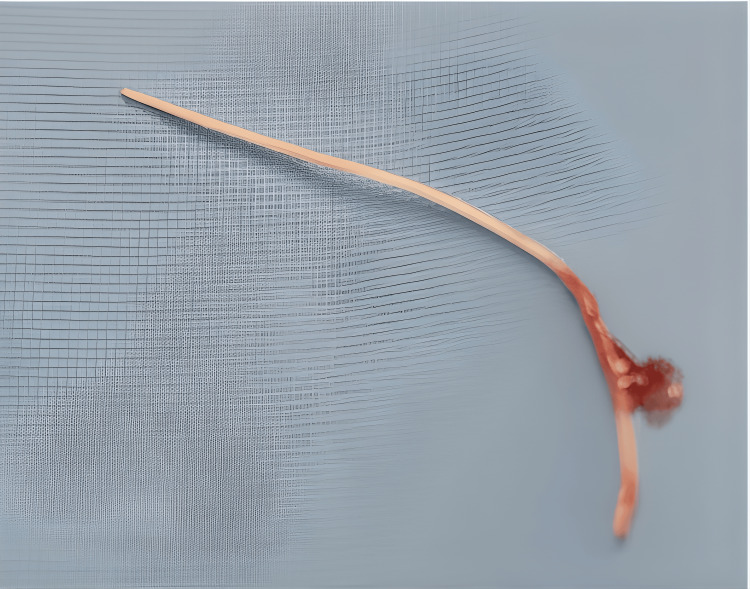
Fish bone foreign body that was removed from the left pyriform sinus of a patient who presented with persistent odynophagia and foreign body sensation.

A 67-year-old woman presented to the ED with odynophagia and persistent foreign body sensation after eating catfish. A neck CT with contrast was ordered and demonstrated no foreign body (Figure 1). She was discharged from the ED on two separate occasions within 24 hours based on negative imaging findings despite ongoing symptoms. The initial physical exam was limited due to the patient’s intolerance of the oral digital examination. The patient was then sedated under general anesthesia, and an oral digital examination was performed. The tongue was retracted, which revealed inflamed tissue and a small object in the left pyriform sinus. Further examination demonstrated approximately 4 cm of a thin, linear fish bone that was embedded within the left pyriform sinus (Figure 2). The bone was then extracted with a hemostat. A flexible endoscope was subsequently advanced to assess for any residual damage to the gastrointestinal tract. Following the procedure, the patient had immediate resolution of her symptoms and recovered without any complications.

## Discussion

The pyriform sinus is a paired hypopharyngeal recess located on each side of the laryngeal inlet and is a common site of foreign body impaction, along with the palatine tonsils and base of the tongue [3,4,7]. Strong contractions of the hypopharyngeal and cricoesophageal musculature mediate the deposition of sharp foreign bodies into the oropharyngeal mucosa, increasing the risk for impaction or perforation. Sharp, linear fish bones are more commonly seen impacted in the pharynx and have a higher risk of local damage. In contrast, flat or geometric bones are more prone to impact within the esophagus. Impaction within the esophagus is less common overall and observed more frequently in individuals >40 years old [4].

Radiologic evaluation of fish bone foreign bodies includes plain film lateral X-ray, CT scan, or flexible fiberoptic laryngoscopy (FFL). False negative rates of plain film X-ray for fish bone identification have been reported to be as high as 85% [2,14]. CT scan has reported sensitivities ranging between 83.3%-100%, and specificities ranging between 93.7% and 100% [11-13]. However, as seen in this case, CT may occasionally miss a fish bone foreign body for two main reasons: either the bone is deeply impacted within the mucosa or the CT slice thickness is too great to detect it [11]. Section thickness is a key technical limitation, as slices greater than 1.5 mm may obscure small or minimally calcified foreign bodies and increase the likelihood of missed lesions. Thinner slice acquisition, ideally 1.5 mm or less, improves spatial resolution and enhances detection of subtle linear foreign bodies, thereby improving diagnostic accuracy [6,11]. FFL alone was found to have a sensitivity of 25%, and is thus typically used in conjunction with other diagnostic methods [11]. 

Oral examination is the best initial step in the evaluation of fish bone foreign bodies. Most fish bones are lodged in the oral cavity, or oropharynx, and many can be removed directly [2,3]. Lower fish bone foreign bodies, however, may require endoscopic removal, typically with the use of a flexible endoscope first. Rigid laryngoscopy or surgical intervention is recommended if difficulty with flexible endoscopy is encountered to avoid further complications [3]. Fish bones lodged in the hypopharynx are associated with higher complication rates, and endoscopy in this region tends to be higher risk [2-4]. Subsequent evaluation for fish bone foreign bodies with esophagogastroduodenoscopy (EGD) is recommended (preferably within two hours, but at latest within six hours) for removal of sharp-pointed foreign bodies to evaluate for esophageal perforation, which has an incidence as high as 35% [2]. For deep or submucosal bones in the pyriform sinus, transoral robotic surgery (TORS) has been proposed as an additional removal method in recent studies [5].

If left untreated, fish bones can migrate into the soft tissues of the neck or thyroid gland. Additionally, oral intake may worsen the impaction, increasing the risk of medical emergencies such as perforation of the esophagus and surrounding structures. Other common complications from fish bone impaction include perforation, abscess formation, deep lacerations with bleeding, and, in severe cases, sepsis, deep neck, and mediastinal abscesses [8-10]. Delayed removal significantly increases complication risk. Previous studies have shown that endoscopic retrieval greater than 24 hours after impaction increases complications fourfold, and greater than 48 hours increases risk by 8.5-fold [8,10].

## Conclusions

This case highlights that fish bones may be radiographically occult, emphasizing the importance of a thorough examination based on the patient's clinical symptoms. Clinical judgment should supersede negative imaging findings to avoid dangerous complications and improve patient outcomes. Emergency physicians and otolaryngologists should maintain a high index of suspicion when clinical presentation strongly suggests fish bone foreign body impaction.
